# 2,4,5-Tris(4-methyl­phen­yl)-1*H*-imidazol-3-ium 2,4,6-tri­nitro­phenolate

**DOI:** 10.1107/S2414314626006711

**Published:** 2026-06-30

**Authors:** Peter Solo, Michael Pillay, Tharsius Raja William Raja

**Affiliations:** a Department of Chemistry, St. Joseph’s College (A), Jakhama, Nagaland, 797001, India; b Department of Environmental Studies, St. Xavier College, Jalukie, Nagaland, India; c Department of Life and Consumer Sciences, College of Agriculture and Environmental Sciences, Florida Campus, University of South Africa, Johannesburg 1709, South Africa; d Postgraduate and Research Department of Biotechnology, Bishop Heber College (Autonomous) Tiruchirappalli Tamil Nadu - 620 017 India; Goethe-Universität Frankfurt, Germany

**Keywords:** crystal structure, imidazolium picrate, π–π stacking

## Abstract

The title mol­ecular salt, C_24_H_23_N_2_^+.^C_6_H_2_N_3_O_7_, consists of a 2,4,5-tris­(4-methyl­phen­yl)-1*H*-imidazol-3-ium cation and a 2,4,6-tri­nitro­phenolate anion. The compound crystallizes in the monoclinic space group *P*2_1_/*c*. In the crystal, the cation and anion are linked by N—H⋯O hydrogen bonds. One imidazolium N—H group forms a hydrogen bond to the phenolate oxygen atom of the picrate anion, while the second N—H group forms an inter­molecular hydrogen bond to a disordered nitro oxygen atom of a symmetry-related picrate anion. The crystal packing is further consolidated by several offset π–π stacking contacts between neighbouring aromatic rings, with centroid–centroid separations in the range 3.560 (2)–3.872 (2) Å. The oxygen atoms of the nitro groups of the picrate anion are disordered over two sets of positions and were refined with split occupancies using suitable geometric and displacement-parameter restraints.

## Abstract 2

The crystal structure of the title imidazolium picrate salt is consolidated by N—H⋯O hydrogen bonds and multiple offset π–π stacking contacts between neighbouring aromatic rings; the nitro-group oxygen atoms of the picrate anion are positionally disordered.

## Graphical abstract

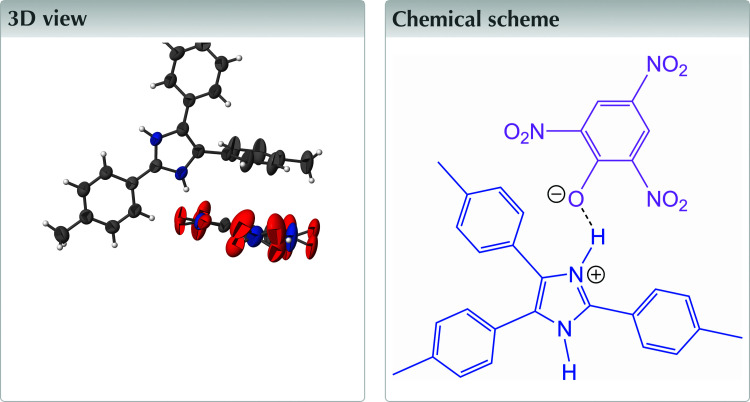

## Structure description

**Figure 1:**
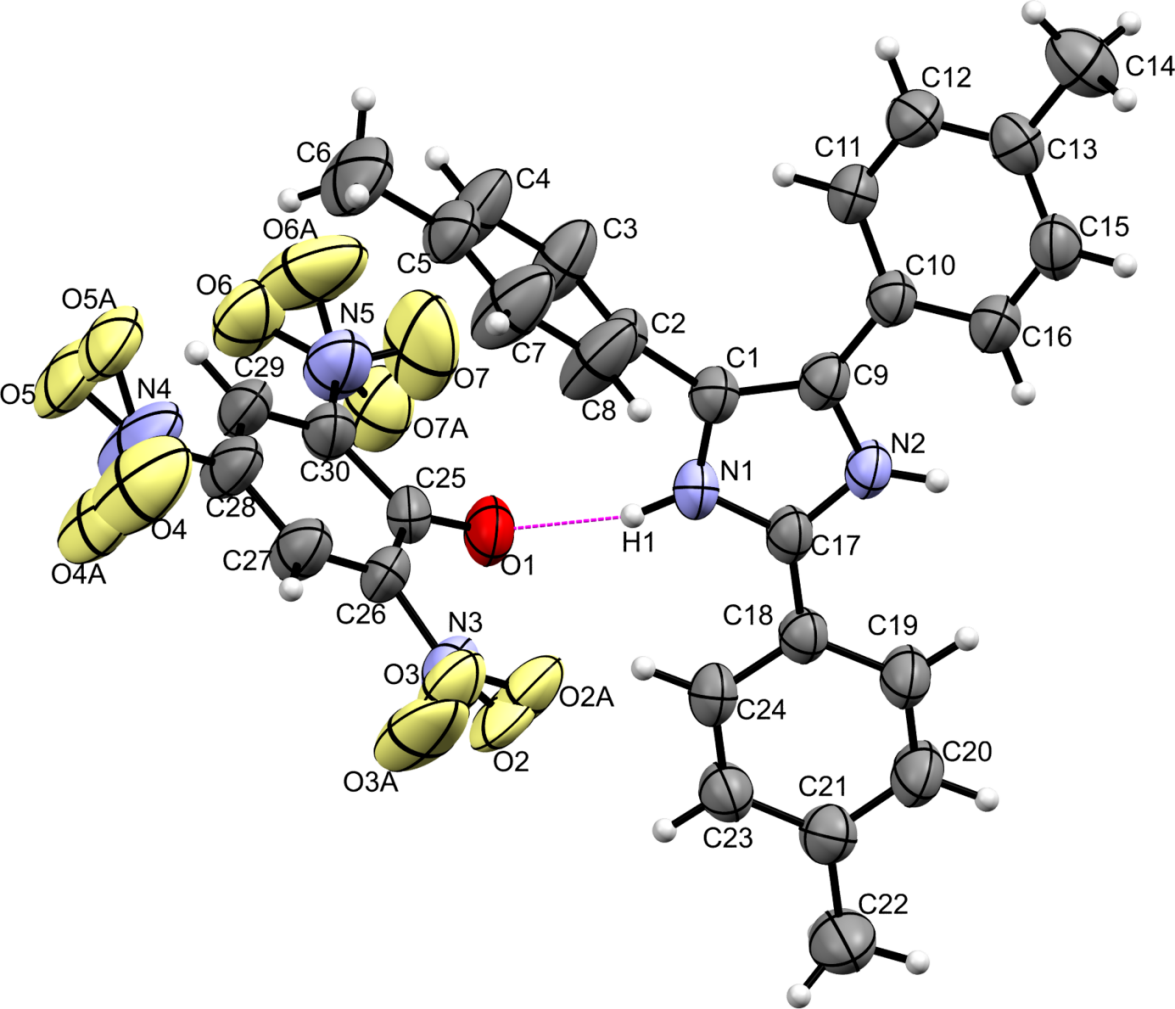
The asymmetric unit of the title compound, showing the 2,4,5-tris­(4-methyl­phen­yl)-1*H*-imidazol-3-ium cation and the 2,4,6-tri­nitro­phenolate anion. Displacement ellipsoids are drawn at the 50% probability level. The N—H⋯O hydrogen-bonding inter­action is shown as a magenta dashed line. The disordered nitro-group oxygen atoms (yellow) are shown over two positions.

**Figure 2:**
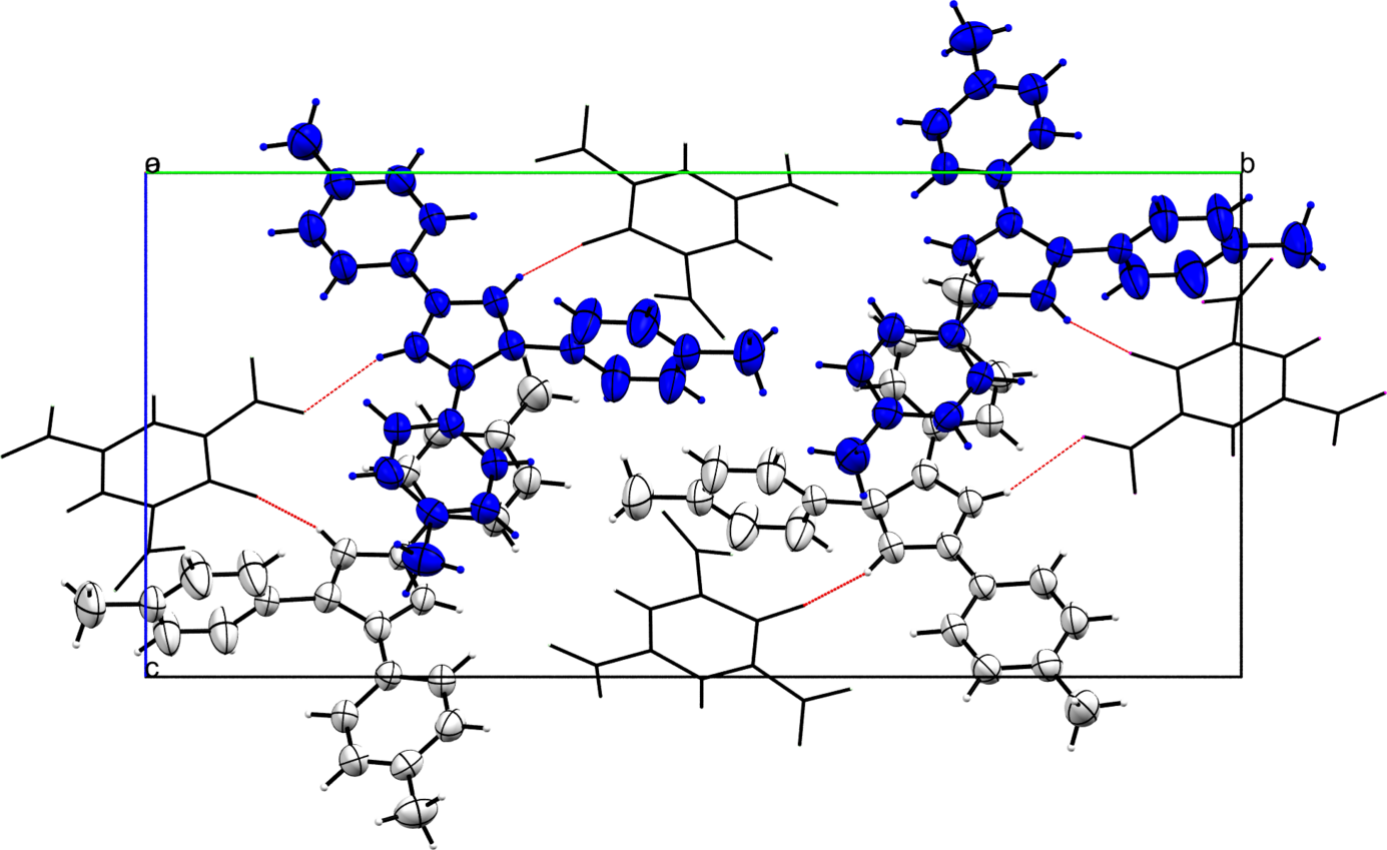
Unit-cell packing diagram of the title compound viewed along the *a* axis. The picrate anions are shown in capped-stick representation, while the imidazolium cations are shown in ellipsoid representation. Hydrogen-bonding inter­actions are indicated by red dashed lines.

Picric acid is a strong proton donor that forms crystalline proton-transfer salts with basic N-heterocycles such as imidazoles, and several imidazolium picrate structures have been reported, including a closely related 2-phenyl-4,5-bis(4-methylphenyl) analogue (Solo *et al*., 2025 ▸). The asymmetric unit of the title compound contains one 2,4,5-tris­(4-methyl­phen­yl)-1*H*-imidazol-3-ium cation and one 2,4,6-tri­nitro­phenolate anion (Fig. 1 ▸). The cation contains an imidazolium ring substituted by three 4-methyl­phenyl groups, while the anion corresponds to the deprotonated form of picric acid. The unit cell consists of four cation–anion pairs as shown in Fig. 2 ▸.

**Figure 3:**
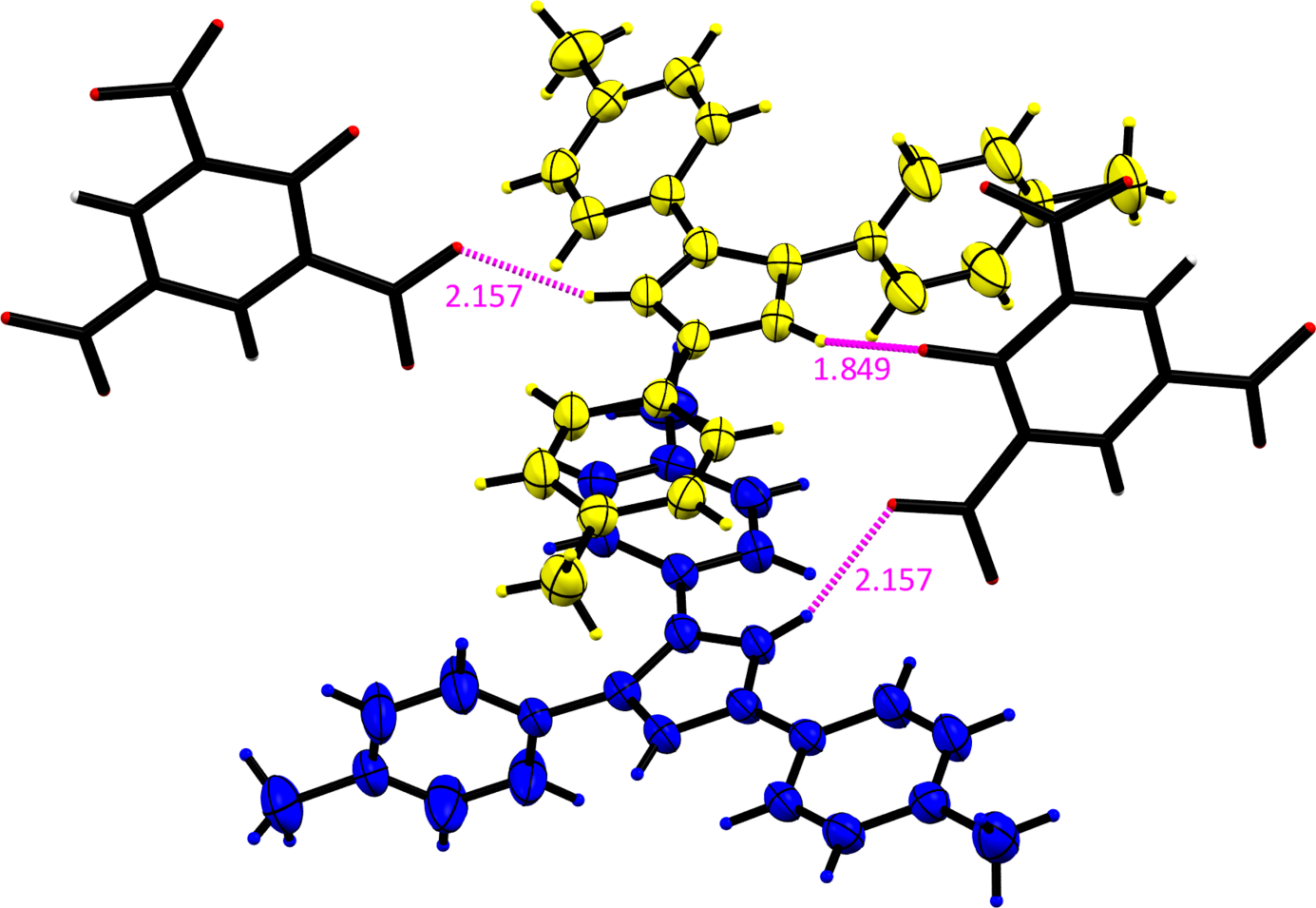
Hydrogen-bonding inter­actions in the crystal structure. The picrate anions are shown in capped-stick representation, while the imidazolium cation is shown in ellipsoid representation (cations coloured as blue and yellow for clarity). The hydrogen-bonding inter­actions are indicated by magenta dashed lines, and the corresponding H⋯O distances (Å) are given.

The cation and anion are associated through an N—H⋯O hydrogen bond involving the imidazolium N—H donor N1—H1 and the phenolate oxygen atom O1 of the picrate anion (Table 1 ▸, Fig. 3 ▸). A second imidazolium N—H group, N2—H2, is involved in an inter­molecular hydrogen bond to the disordered nitro oxygen atom O2/O2*A* of a symmetry-related picrate anion. Since O2 and O2*A* are alternative disorder positions, these contacts represent alternative hydrogen-bonding inter­actions involving the same disordered nitro group.

**Figure 4:**
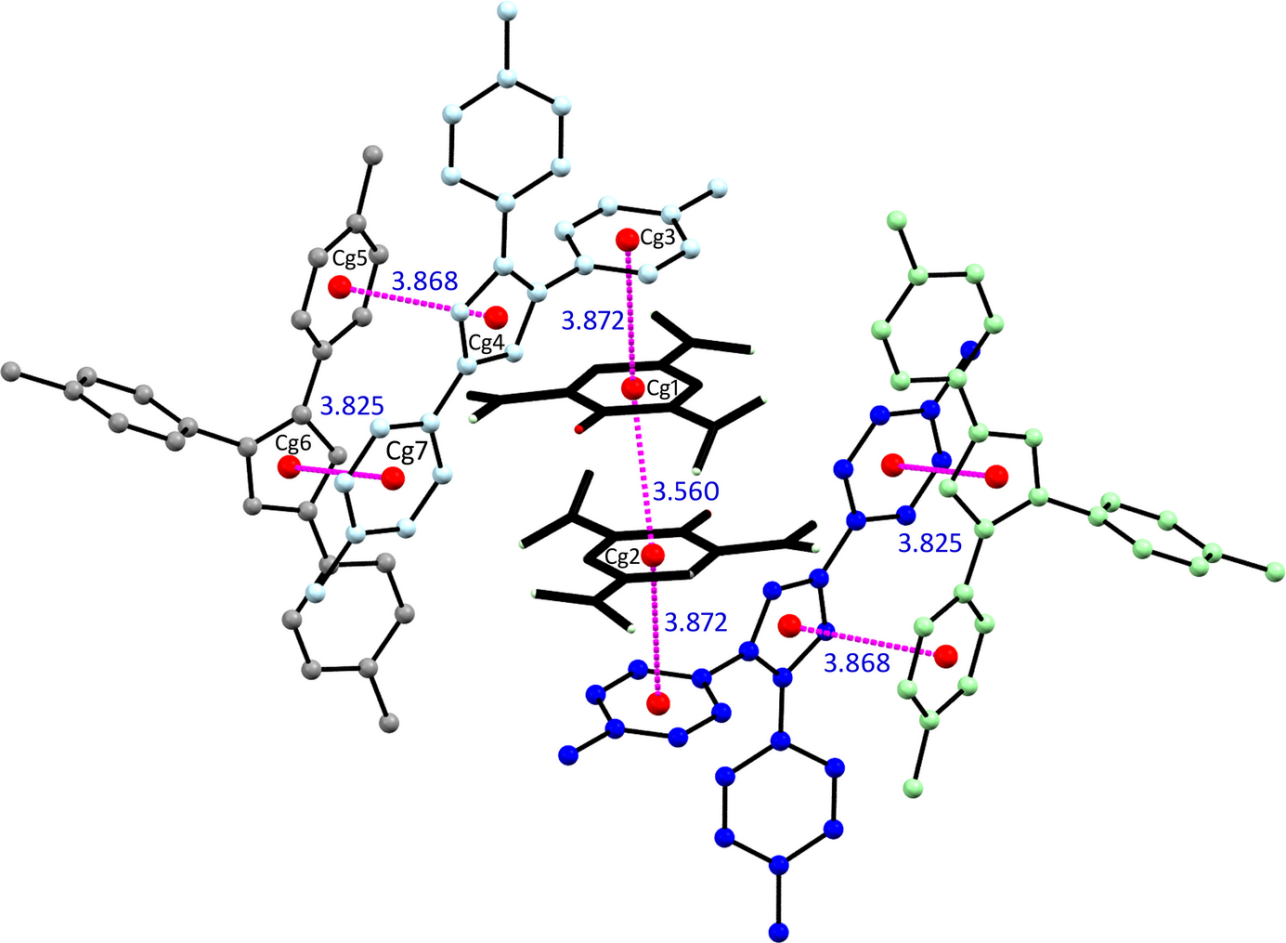
Packing diagram showing π–π stacking inter­actions between neighbouring aromatic rings. The picrate anions are shown in capped-stick representation, while the imidazolium cations are shown in ball-and-stick representation, with each cations displayed in different colours for clarity. Ring centroids are shown as red spheres, and centroid–centroid contacts are represented by magenta dashed lines, with centroid distances given in Å. Hydrogen atoms have been omitted for clarity.

The crystal packing is further supported by several offset π–π stacking contacts between neighbouring aromatic rings (Fig. 4 ▸). The centroid–centroid separations lie in the range 3.560 (2)–3.872 (2) Å, with perpendicular inter­planar separations of 3.435–3.746 Å, inter­planar angles of 0.00–13.38° and slippage values of 0.935–1.317 Å (Table 2 ▸). These values are consistent with weak to moderate offset π–π stacking inter­actions (Martinez & Iverson, 2012 ▸; Janiak, 2000 ▸).

The nitro groups of the tri­nitro­phenolate anion show positional disorder. The oxygen atoms of the nitro groups attached at C26, C28 and C30 were modelled over two positions. The refined occupancies are 0.41 (4)/0.59 (4) for the O2/O3 and O2*A*/O3*A* components, 0.75 (3)/0.25 (3) for the O4/O5 and O4*A*/O5*A* components, and 0.413 (18)/0.587 (18) for the O6/O7 and O6*A*/O7*A* components.

## Synthesis and crystallization

**Figure 5:**
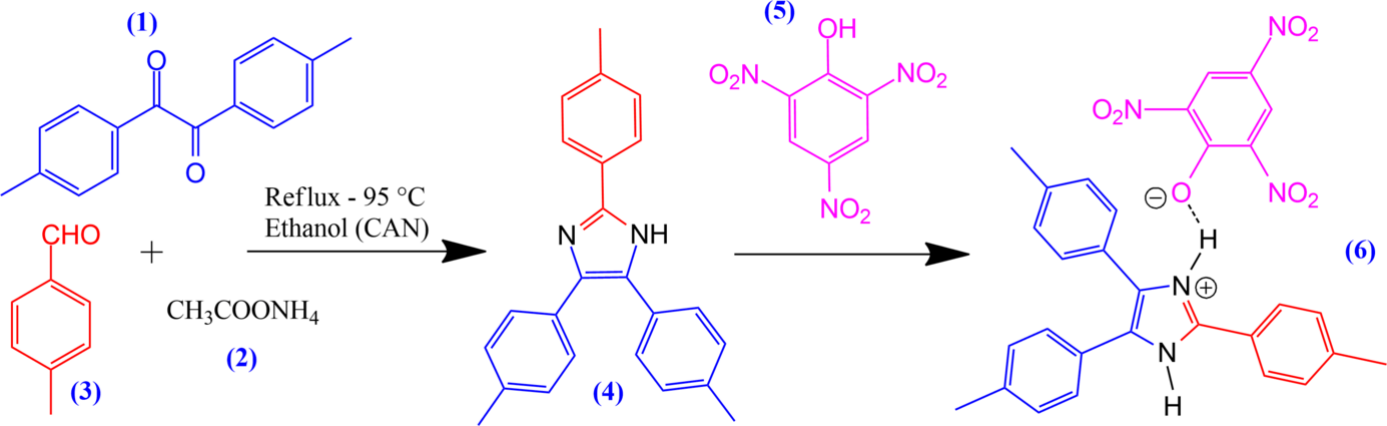
Synthesis of the title imidazolium picrate salt from 4,4′-di­methyl­benzil (**1**), ammonium acetate (**2**), 4-methyl­benzaldehyde (**3**) and picric acid (**5**). The imidazole inter­mediate (**4**) was obtained in ethanol/CAN under reflux at 95°C, followed by salt formation with picric acid.

2,4,5-Tris(4-methyl­phen­yl)-1*H*-imidazole (**4**) was synthesized by a one-pot condensation reaction of 4,4′-di­methyl­benzil (**1**) (0.953 g, 0.004 mol), 4-methyl­benzaldehyde (**3**) (0.481 g, 0.004 mol) and ammonium acetate (**2**) (1.233 g, 0.016 mol) in the presence of ceric ammonium nitrate (CAN) as catalyst. Ethanol was used as the solvent and the reflux was carried out at 95°C. The progress of the reaction was monitored by TLC using hexa­ne:ethyl acetate (1:1) as eluent. After completion of the reaction, the mixture was poured into ice-cold water. The precipitate obtained was collected and purified by repeated recrystallization from 90% ethanol solution. Equimolar amounts of 2,4,5-tris­(4-methyl­phen­yl)-1*H*-imidazole (0.068 g, 0.0002 mol) and picric acid (0.046 g, 0.0002 mol) were dissolved in 100% ethanol and heated to 120°C (Fig. 5 ▸). The solution was kept undisturbed in the dark for several days until clear yellowish-orange crystals of the title salt were obtained.

## Refinement

Crystal data, data collection and structure refinement details are summarized in the crystallographic data Table 3 ▸. Hydrogen atoms were placed in calculated positions and refined using a riding model, with *U*_iso_(H) values constrained to appropriate multiples of the equivalent isotropic displacement parameters of their parent atoms. Methyl hydrogen atoms were treated as rotating groups.

The oxygen atoms of the nitro groups were modelled over two sets of positions and refined with split occupancies. Equivalent N—O and O⋯O distances in the disordered nitro groups were restrained using SADI restraints. Softer SADI restraints with an effective standard uncertainty of 0.04 Å were used for the more disordered O6/O7 and O6A/O7A nitro-group components, while the remaining equivalent nitro-group distances were restrained using the default SADI value. The nitro groups were restrained to be approximately planar using FLAT restraints; a softer FLAT restraint with an effective standard uncertainty of 0.2 Å was applied to the more disordered N5/O6/O7 group. The anisotropic displacement parameters of the disordered atoms were restrained using SIMU and RIGU restraints.

The final refinement gave *R*1 = 0.0682 for observed reflections, *wR*2 = 0.1995 for all data and *S* = 1.012. The relatively high *R*_int_ value and the displacement-parameter alerts are attributed mainly to weak high-angle diffraction and the positional disorder of the nitro-group oxygen atoms in the picrate anion.

## Supplementary Material

Crystal structure: contains datablock(s) I. DOI: 10.1107/S2414314626006711/bt4202sup1.cif

Structure factors: contains datablock(s) I. DOI: 10.1107/S2414314626006711/bt4202Isup2.hkl

Supporting information file. DOI: 10.1107/S2414314626006711/bt4202Isup3.cml

CCDC reference: 2559623

Additional supporting information:  crystallographic information; 3D view; checkCIF report

## Figures and Tables

**Table 1:** Hydrogen-bond geometry (Å, °)

*D*—H⋯*A*	*D*—H	H⋯*A*	*D*⋯*A*	*D*—H⋯*A*
N1—H1⋯O1	0.86	1.85	2.684 (3)	163
N2—H2⋯O2^i^	0.86	2.09	2.927 (17)	165
N2—H2⋯O2*A*^i^	0.86	2.16	2.974 (11)	158

Symmetry code: (i) 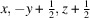.

**Table 2:** π–π stacking parameters (Å, °) for the title compound

Contact	Ring centroids	*Cg*⋯*Cg* distance	Perpendicular distance	Inter­planar angle	Slippage
1	*Cg*1⋯*Cg*2	3.560 (2)	3.435	0.00	0.935
2	*Cg*1⋯*Cg*3	3.872 (2)	3.746	12.11	0.980
3	*Cg*4⋯*Cg*5	3.868 (2)	3.690	11.95	1.160
4	*Cg*6⋯*Cg*7	3.825 (2)	3.591	13.38	1.317

*Cg*1–*Cg*7 are the centroids of the corresponding aromatic rings shown in Fig. 4 ▸.

**Table 3:** Experimental details

Crystal data	
Chemical formula	C_24_H_23_N_2_^+^·C_6_H_2_N_3_O_7_^−^
*M*_r_	567.55
Crystal system, space group	Monoclinic, *P*2_1_/*c*
Temperature (K)	296
*a*, *b*, *c* (Å)	9.6095 (15), 24.877 (4), 11.4691 (18)
β (°)	92.686 (5)
*V* (Å^3^)	2738.8 (7)
*Z*	4
Radiation type	Mo *K*α
μ (mm^−1^)	0.10
Crystal size (mm)	0.2 × 0.12 × 0.08

Data collection	
Diffractometer	Bruker APEXII CCD
Absorption correction	Multi-scan (*SADABS*; Krause *et al.*, 2015 ▸)
*T*_min_, *T*_max_	0.509, 0.746
No. of measured, independent and observed [*I* > 2σ(*I*)] reflections	121811, 7135, 3162
*R*_int_	0.157
(sin θ/λ)_max_ (Å^−1^)	0.678

Refinement	
*R*[*F*^2^ > 2σ(*F*^2^)], *wR*(*F*^2^), *S*	0.068, 0.200, 1.01
No. of reflections	7135
No. of parameters	440
No. of restraints	189
H-atom treatment	H-atom parameters constrained
Δρ_max_, Δρ_min_ (e Å^−3^)	0.26, −0.21

Computer programs: *APEX2* and *SAINT* (Bruker, 2018 ▸), *SHELXT2018/2* (Sheldrick, 2015*a* ▸), *SHELXL2025/1* (Sheldrick, 2015*b* ▸), *OLEX2* (Dolomanov *et al.*, 2009 ▸) and *Mercury* (Macrae *et al.*, 2020 ▸).

## full crystallographic data

### 2,4,5-Tris(4-methylphenyl)-1H-imidazol-3-ium 2,4,6-trinitrophenolate Crystal data

**Table:**

C_24_H_23_N_2_^+^·C_6_H_2_N_3_O_7_^−^	*F*(000) = 1184
*M**_r_* = 567.55	*D*_x_ = 1.376 Mg m^−^^3^
Monoclinic, *P*2_1_/*c*	Mo *K*α radiation, λ = 0.71073 Å
*a* = 9.6095 (15) Å	Cell parameters from 5170 reflections
*b* = 24.877 (4) Å	θ = 2.3–18.6°
*c* = 11.4691 (18) Å	µ = 0.10 mm^−^^1^
β = 92.686 (5)°	*T* = 296 K
*V* = 2738.8 (7) Å^3^	Irregular, clear yellowish orange
*Z* = 4	0.2 × 0.12 × 0.08 mm

### 2,4,5-Tris(4-methylphenyl)-1H-imidazol-3-ium 2,4,6-trinitrophenolate Data collection

**Table:**

Bruker APEXII CCD diffractometer	3162 reflections with *I* > 2σ(*I*)
φ and ω scans	*R*_int_ = 0.157
Absorption correction: multi-scan (SADABS; Krause *et al.*, 2015)	θ_max_ = 28.8°, θ_min_ = 1.6°
*T*_min_ = 0.509, *T*_max_ = 0.746	*h* = −13→12
121811 measured reflections	*k* = −33→33
7135 independent reflections	*l* = −15→15

### 2,4,5-Tris(4-methylphenyl)-1H-imidazol-3-ium 2,4,6-trinitrophenolate Refinement

**Table:**

Refinement on *F*^2^	Hydrogen site location: inferred from neighbouring sites
Least-squares matrix: full	H-atom parameters constrained
*R*[*F*^2^ > 2σ(*F*^2^)] = 0.068	*w* = 1/[σ^2^(*F*_o_^2^) + (0.0669*P*)^2^ + 1.4232*P*] where *P* = (*F*_o_^2^ + 2*F*_c_^2^)/3
*wR*(*F*^2^) = 0.200	(Δ/σ)_max_ < 0.001
*S* = 1.01	Δρ_max_ = 0.26 e Å^−^^3^
7135 reflections	Δρ_min_ = −0.21 e Å^−^^3^
440 parameters	Extinction correction: SHELXL2025/1 (Sheldrick, 2015*b*), Fc^*^=kFc[1+0.001xFc^2^λ^3^/sin(2θ)]^-1/4^
189 restraints	Extinction coefficient: 0.0093 (11)
Primary atom site location: dual	

### 2,4,5-Tris(4-methylphenyl)-1H-imidazol-3-ium 2,4,6-trinitrophenolate Special details

**Table:**

Geometry. All esds (except the esd in the dihedral angle between two l.s. planes) are estimated using the full covariance matrix. The cell esds are taken into account individually in the estimation of esds in distances, angles and torsion angles; correlations between esds in cell parameters are only used when they are defined by crystal symmetry. An approximate (isotropic) treatment of cell esds is used for estimating esds involving l.s. planes.
Refinement. The oxygen atoms of the nitro (NO2) groups were disordered over two positions and were refined with split occupancies using SADI, SIMU, RIGU and FLAT restraints. An extinction correction was applied.

### 2,4,5-Tris(4-methylphenyl)-1H-imidazol-3-ium 2,4,6-trinitrophenolate Fractional atomic coordinates and isotropic or equivalent isotropic displacement parameters (Å^2^)

**Table:**

	*x*	*y*	*z*	*U*_iso_*/*U*_eq_	Occ. (<1)
C1	0.2696 (3)	0.33411 (10)	0.3426 (2)	0.0485 (7)	
C2	0.2157 (3)	0.38940 (9)	0.3497 (2)	0.0478 (6)	
C3	0.2866 (4)	0.42912 (12)	0.4082 (3)	0.0817 (11)	
H3	0.371368	0.421484	0.447157	0.098*	
C4	0.2337 (4)	0.48068 (12)	0.4104 (4)	0.0903 (12)	
H4	0.284311	0.507152	0.450913	0.108*	
C5	0.1108 (4)	0.49403 (11)	0.3555 (3)	0.0686 (9)	
C6	0.0546 (5)	0.55087 (13)	0.3567 (4)	0.1052 (14)	
H6A	0.053473	0.563451	0.435797	0.158*	
H6B	−0.038343	0.551362	0.322234	0.158*	
H6C	0.113143	0.573833	0.312911	0.158*	
C7	0.0416 (4)	0.45434 (14)	0.2984 (4)	0.1044 (15)	
H7	−0.043322	0.462076	0.259892	0.125*	
C8	0.0923 (4)	0.40240 (13)	0.2949 (4)	0.0942 (13)	
H8	0.041062	0.376069	0.254506	0.113*	
C9	0.2379 (3)	0.28834 (10)	0.4009 (2)	0.0482 (6)	
C10	0.1474 (3)	0.27860 (10)	0.4974 (2)	0.0468 (6)	
C11	0.1228 (3)	0.31836 (11)	0.5783 (2)	0.0548 (7)	
H11	0.168723	0.351164	0.573873	0.066*	
C12	0.0311 (3)	0.30989 (11)	0.6653 (3)	0.0590 (8)	
H12	0.016518	0.337142	0.718852	0.071*	
C13	−0.0397 (3)	0.26177 (12)	0.6747 (2)	0.0540 (7)	
C14	−0.1432 (3)	0.25337 (15)	0.7667 (3)	0.0774 (10)	
H14A	−0.097339	0.237557	0.834531	0.116*	
H14B	−0.215802	0.229838	0.737290	0.116*	
H14C	−0.182682	0.287322	0.787252	0.116*	
C15	−0.0126 (3)	0.22186 (12)	0.5959 (3)	0.0597 (8)	
H15	−0.057046	0.188837	0.601705	0.072*	
C16	0.0792 (3)	0.22968 (10)	0.5082 (3)	0.0551 (7)	
H16	0.095394	0.202014	0.456020	0.066*	
C17	0.3806 (3)	0.26646 (10)	0.2588 (2)	0.0460 (6)	
C18	0.4640 (3)	0.23584 (10)	0.1801 (2)	0.0452 (6)	
C19	0.4735 (3)	0.18060 (11)	0.1879 (3)	0.0594 (8)	
H19	0.427257	0.162529	0.245629	0.071*	
C20	0.5505 (3)	0.15202 (11)	0.1112 (3)	0.0642 (8)	
H20	0.556850	0.114879	0.118899	0.077*	
C21	0.6186 (3)	0.17716 (11)	0.0227 (3)	0.0570 (7)	
C22	0.7009 (4)	0.14518 (14)	−0.0619 (3)	0.0815 (10)	
H22A	0.673084	0.155306	−0.140297	0.122*	
H22B	0.683570	0.107543	−0.051156	0.122*	
H22C	0.798446	0.152331	−0.048005	0.122*	
C23	0.6091 (3)	0.23262 (11)	0.0160 (2)	0.0568 (7)	
H23	0.654905	0.250611	−0.042026	0.068*	
C24	0.5340 (3)	0.26170 (11)	0.0927 (2)	0.0529 (7)	
H24	0.529783	0.298937	0.086205	0.063*	
C25	0.4194 (3)	0.44175 (10)	0.1113 (2)	0.0514 (7)	
C26	0.3083 (3)	0.44583 (10)	0.0226 (2)	0.0549 (7)	
C27	0.2381 (4)	0.49252 (11)	−0.0039 (3)	0.0693 (9)	
H27	0.164654	0.492616	−0.059711	0.083*	
C28	0.2768 (4)	0.53884 (12)	0.0523 (3)	0.0760 (10)	
C29	0.3850 (4)	0.53937 (11)	0.1346 (3)	0.0719 (9)	
H29	0.411117	0.571366	0.171398	0.086*	
C30	0.4540 (3)	0.49316 (11)	0.1622 (2)	0.0597 (8)	
N1	0.3571 (2)	0.31920 (8)	0.25642 (19)	0.0498 (6)	
H1	0.391842	0.341067	0.207535	0.060*	
N2	0.3078 (2)	0.24713 (8)	0.34641 (18)	0.0476 (5)	
H2	0.304788	0.213817	0.366185	0.057*	
N3	0.2692 (3)	0.39936 (10)	−0.0469 (3)	0.0700 (7)	
N4	0.2065 (5)	0.58864 (12)	0.0228 (4)	0.1276 (16)	
N5	0.5676 (4)	0.49712 (13)	0.2506 (3)	0.0858 (9)	
O1	0.4793 (2)	0.39910 (7)	0.14185 (18)	0.0655 (6)	
O2	0.341 (2)	0.3612 (7)	−0.057 (2)	0.098 (6)	0.41 (4)
O3	0.1482 (11)	0.4006 (5)	−0.086 (2)	0.089 (5)	0.41 (4)
O2A	0.3265 (15)	0.3563 (4)	−0.0238 (17)	0.086 (4)	0.59 (4)
O3A	0.195 (3)	0.4031 (5)	−0.1348 (18)	0.152 (6)	0.59 (4)
O4	0.0930 (15)	0.5849 (4)	−0.0385 (15)	0.158 (5)	0.75 (3)
O5	0.2618 (14)	0.6313 (2)	0.0633 (11)	0.117 (3)	0.75 (3)
O4A	0.162 (4)	0.5939 (10)	−0.0823 (12)	0.115 (8)	0.25 (3)
O5A	0.187 (3)	0.6231 (8)	0.1026 (14)	0.095 (6)	0.25 (3)
O6	0.6143 (13)	0.5448 (3)	0.2643 (13)	0.104 (4)	0.413 (18)
O7	0.594 (2)	0.4619 (4)	0.3131 (17)	0.148 (7)	0.413 (18)
O6A	0.5599 (13)	0.5279 (6)	0.3283 (11)	0.146 (5)	0.587 (18)
O7A	0.6676 (7)	0.4652 (3)	0.2436 (9)	0.096 (3)	0.587 (18)

### 2,4,5-Tris(4-methylphenyl)-1H-imidazol-3-ium 2,4,6-trinitrophenolate Atomic displacement parameters (Å^2^)

**Table:**

	*U*^11^	*U*^22^	*U*^33^	*U*^12^	*U*^13^	*U*^23^
C1	0.0559 (16)	0.0389 (14)	0.0505 (16)	0.0038 (12)	0.0006 (13)	0.0037 (12)
C2	0.0610 (17)	0.0331 (13)	0.0494 (16)	0.0035 (12)	0.0018 (13)	0.0029 (11)
C3	0.074 (2)	0.0450 (17)	0.123 (3)	0.0065 (15)	−0.029 (2)	−0.0080 (18)
C4	0.098 (3)	0.0400 (17)	0.130 (3)	0.0010 (17)	−0.026 (2)	−0.0178 (19)
C5	0.094 (3)	0.0395 (16)	0.071 (2)	0.0157 (15)	−0.0025 (19)	−0.0012 (14)
C6	0.144 (4)	0.053 (2)	0.117 (3)	0.035 (2)	−0.009 (3)	−0.006 (2)
C7	0.114 (3)	0.064 (2)	0.129 (3)	0.035 (2)	−0.059 (3)	−0.022 (2)
C8	0.102 (3)	0.0530 (19)	0.122 (3)	0.0168 (18)	−0.058 (2)	−0.025 (2)
C9	0.0515 (16)	0.0380 (13)	0.0547 (16)	0.0039 (11)	−0.0027 (13)	0.0017 (12)
C10	0.0526 (16)	0.0377 (14)	0.0498 (16)	0.0034 (11)	−0.0003 (13)	0.0034 (11)
C11	0.0703 (19)	0.0378 (14)	0.0566 (17)	−0.0021 (13)	0.0055 (15)	0.0020 (12)
C12	0.073 (2)	0.0498 (17)	0.0546 (18)	0.0070 (14)	0.0050 (16)	−0.0017 (13)
C13	0.0547 (17)	0.0582 (18)	0.0486 (16)	0.0008 (13)	−0.0010 (13)	0.0110 (13)
C14	0.068 (2)	0.100 (3)	0.064 (2)	−0.0071 (19)	0.0089 (17)	0.0106 (19)
C15	0.0679 (19)	0.0497 (17)	0.0612 (19)	−0.0116 (14)	−0.0008 (16)	0.0101 (14)
C16	0.0681 (19)	0.0394 (14)	0.0577 (18)	−0.0008 (13)	0.0011 (15)	0.0002 (12)
C17	0.0525 (16)	0.0365 (13)	0.0484 (15)	0.0018 (11)	−0.0033 (13)	0.0075 (11)
C18	0.0485 (15)	0.0401 (14)	0.0467 (15)	0.0021 (11)	−0.0008 (12)	0.0048 (11)
C19	0.073 (2)	0.0407 (15)	0.0659 (19)	0.0052 (14)	0.0160 (16)	0.0086 (13)
C20	0.077 (2)	0.0406 (15)	0.076 (2)	0.0078 (14)	0.0131 (18)	0.0066 (14)
C21	0.0626 (18)	0.0526 (17)	0.0557 (17)	0.0079 (14)	0.0024 (15)	0.0030 (14)
C22	0.100 (3)	0.068 (2)	0.078 (2)	0.0174 (19)	0.024 (2)	0.0008 (18)
C23	0.0638 (19)	0.0527 (17)	0.0545 (17)	0.0033 (14)	0.0087 (15)	0.0091 (13)
C24	0.0591 (17)	0.0405 (14)	0.0588 (17)	0.0028 (12)	0.0008 (14)	0.0094 (13)
C25	0.0700 (19)	0.0350 (14)	0.0499 (16)	−0.0035 (12)	0.0107 (14)	0.0082 (12)
C26	0.077 (2)	0.0311 (13)	0.0569 (17)	−0.0034 (12)	0.0027 (15)	−0.0038 (12)
C27	0.091 (2)	0.0476 (17)	0.067 (2)	0.0104 (16)	−0.0176 (18)	−0.0040 (15)
C28	0.118 (3)	0.0400 (16)	0.068 (2)	0.0220 (17)	−0.022 (2)	−0.0055 (14)
C29	0.117 (3)	0.0372 (15)	0.0599 (19)	0.0009 (16)	−0.0107 (19)	−0.0047 (13)
C30	0.084 (2)	0.0445 (16)	0.0499 (17)	−0.0048 (14)	−0.0075 (16)	0.0027 (13)
N1	0.0612 (14)	0.0349 (11)	0.0532 (14)	0.0013 (10)	0.0025 (11)	0.0086 (10)
N2	0.0556 (13)	0.0328 (11)	0.0543 (13)	0.0022 (9)	0.0013 (11)	0.0058 (10)
N3	0.090 (2)	0.0434 (14)	0.0757 (19)	−0.0070 (14)	−0.0044 (16)	−0.0062 (13)
N4	0.199 (4)	0.055 (2)	0.122 (3)	0.046 (2)	−0.068 (3)	−0.0217 (19)
N5	0.114 (3)	0.0655 (19)	0.075 (2)	−0.0011 (18)	−0.0241 (19)	−0.0013 (16)
O1	0.0785 (14)	0.0419 (11)	0.0769 (14)	0.0045 (10)	0.0110 (11)	0.0134 (10)
O2	0.126 (7)	0.044 (5)	0.127 (13)	0.007 (4)	0.037 (7)	−0.032 (6)
O3	0.110 (6)	0.053 (4)	0.099 (9)	−0.008 (4)	−0.041 (6)	−0.013 (5)
O2A	0.104 (6)	0.045 (3)	0.106 (8)	0.006 (3)	−0.031 (6)	−0.024 (3)
O3A	0.259 (12)	0.084 (5)	0.102 (8)	0.027 (7)	−0.099 (9)	−0.032 (5)
O4	0.202 (8)	0.092 (5)	0.171 (9)	0.065 (5)	−0.096 (8)	−0.024 (5)
O5	0.181 (7)	0.040 (2)	0.127 (6)	0.023 (3)	−0.027 (6)	−0.015 (3)
O4A	0.164 (19)	0.057 (9)	0.117 (8)	0.048 (11)	−0.055 (10)	−0.010 (8)
O5A	0.135 (17)	0.042 (7)	0.108 (9)	0.016 (8)	−0.014 (8)	−0.009 (6)
O6	0.139 (8)	0.073 (4)	0.097 (8)	−0.022 (4)	−0.039 (6)	−0.012 (4)
O7	0.188 (14)	0.107 (6)	0.142 (11)	0.001 (7)	−0.077 (11)	0.049 (6)
O6A	0.177 (8)	0.132 (8)	0.121 (7)	0.034 (6)	−0.066 (6)	−0.062 (6)
O7A	0.100 (4)	0.077 (3)	0.107 (6)	0.006 (3)	−0.034 (4)	0.010 (3)

### 2,4,5-Tris(4-methylphenyl)-1H-imidazol-3-ium 2,4,6-trinitrophenolate Geometric parameters (Å, º)

**Table:**

C1—C2	1.473 (3)	C19—H19	0.9300
C1—C9	1.362 (3)	C19—C20	1.374 (4)
C1—N1	1.378 (3)	C20—H20	0.9300
C2—C3	1.360 (4)	C20—C21	1.383 (4)
C2—C8	1.355 (4)	C21—C22	1.507 (4)
C3—H3	0.9300	C21—C23	1.385 (4)
C3—C4	1.380 (4)	C22—H22A	0.9600
C4—H4	0.9300	C22—H22B	0.9600
C4—C5	1.354 (5)	C22—H22C	0.9600
C5—C6	1.514 (4)	C23—H23	0.9300
C5—C7	1.343 (5)	C23—C24	1.370 (4)
C6—H6A	0.9600	C24—H24	0.9300
C6—H6B	0.9600	C25—C26	1.443 (4)
C6—H6C	0.9600	C25—C30	1.438 (4)
C7—H7	0.9300	C25—O1	1.250 (3)
C7—C8	1.382 (4)	C26—C27	1.370 (4)
C8—H8	0.9300	C26—N3	1.444 (3)
C9—C10	1.459 (4)	C27—H27	0.9300
C9—N2	1.390 (3)	C27—C28	1.364 (4)
C10—C11	1.384 (4)	C28—C29	1.371 (4)
C10—C16	1.391 (4)	C28—N4	1.444 (4)
C11—H11	0.9300	C29—H29	0.9300
C11—C12	1.378 (4)	C29—C30	1.357 (4)
C12—H12	0.9300	C30—N5	1.458 (4)
C12—C13	1.384 (4)	N1—H1	0.8600
C13—C14	1.498 (4)	N2—H2	0.8600
C13—C15	1.376 (4)	N3—O2	1.183 (12)
C14—H14A	0.9600	N3—O3	1.228 (10)
C14—H14B	0.9600	N3—O2A	1.228 (8)
C14—H14C	0.9600	N3—O3A	1.212 (8)
C15—H15	0.9300	N4—O4	1.272 (7)
C15—C16	1.381 (4)	N4—O5	1.265 (6)
C16—H16	0.9300	N4—O4A	1.268 (13)
C17—C18	1.451 (4)	N4—O5A	1.274 (11)
C17—N1	1.331 (3)	N5—O6	1.276 (8)
C17—N2	1.340 (3)	N5—O7	1.153 (9)
C18—C19	1.380 (3)	N5—O6A	1.180 (7)
C18—C24	1.391 (4)	N5—O7A	1.252 (6)
			
C9—C1—C2	131.8 (3)	C20—C19—H19	119.7
C9—C1—N1	106.6 (2)	C19—C20—H20	119.2
N1—C1—C2	121.2 (2)	C19—C20—C21	121.6 (3)
C3—C2—C1	122.4 (3)	C21—C20—H20	119.2
C8—C2—C1	120.0 (3)	C20—C21—C22	121.0 (3)
C8—C2—C3	117.6 (3)	C20—C21—C23	117.3 (3)
C2—C3—H3	119.7	C23—C21—C22	121.7 (3)
C2—C3—C4	120.5 (3)	C21—C22—H22A	109.5
C4—C3—H3	119.7	C21—C22—H22B	109.5
C3—C4—H4	118.9	C21—C22—H22C	109.5
C5—C4—C3	122.3 (3)	H22A—C22—H22B	109.5
C5—C4—H4	118.9	H22A—C22—H22C	109.5
C4—C5—C6	121.9 (3)	H22B—C22—H22C	109.5
C7—C5—C4	116.6 (3)	C21—C23—H23	119.1
C7—C5—C6	121.5 (3)	C24—C23—C21	121.8 (3)
C5—C6—H6A	109.5	C24—C23—H23	119.1
C5—C6—H6B	109.5	C18—C24—H24	119.8
C5—C6—H6C	109.5	C23—C24—C18	120.4 (3)
H6A—C6—H6B	109.5	C23—C24—H24	119.8
H6A—C6—H6C	109.5	C30—C25—C26	111.9 (2)
H6B—C6—H6C	109.5	O1—C25—C26	125.0 (2)
C5—C7—H7	118.8	O1—C25—C30	123.1 (3)
C5—C7—C8	122.3 (3)	C25—C26—N3	119.8 (2)
C8—C7—H7	118.8	C27—C26—C25	123.8 (2)
C2—C8—C7	120.8 (3)	C27—C26—N3	116.3 (3)
C2—C8—H8	119.6	C26—C27—H27	120.3
C7—C8—H8	119.6	C28—C27—C26	119.4 (3)
C1—C9—C10	131.8 (2)	C28—C27—H27	120.3
C1—C9—N2	105.8 (2)	C27—C28—C29	121.0 (3)
N2—C9—C10	122.4 (2)	C27—C28—N4	120.0 (3)
C11—C10—C9	121.1 (2)	C29—C28—N4	119.0 (3)
C11—C10—C16	118.0 (3)	C28—C29—H29	120.0
C16—C10—C9	120.9 (2)	C30—C29—C28	119.9 (3)
C10—C11—H11	119.6	C30—C29—H29	120.0
C12—C11—C10	120.7 (3)	C25—C30—N5	119.6 (3)
C12—C11—H11	119.6	C29—C30—C25	123.9 (3)
C11—C12—H12	119.2	C29—C30—N5	116.5 (3)
C11—C12—C13	121.5 (3)	C1—N1—H1	124.5
C13—C12—H12	119.2	C17—N1—C1	111.0 (2)
C12—C13—C14	121.4 (3)	C17—N1—H1	124.5
C15—C13—C12	117.6 (3)	C9—N2—H2	124.7
C15—C13—C14	121.0 (3)	C17—N2—C9	110.6 (2)
C13—C14—H14A	109.5	C17—N2—H2	124.7
C13—C14—H14B	109.5	O2—N3—C26	123.8 (11)
C13—C14—H14C	109.5	O2—N3—O3	122.3 (11)
H14A—C14—H14B	109.5	O3—N3—C26	113.6 (8)
H14A—C14—H14C	109.5	O2A—N3—C26	118.5 (6)
H14B—C14—H14C	109.5	O3A—N3—C26	121.7 (6)
C13—C15—H15	119.2	O3A—N3—O2A	119.2 (8)
C13—C15—C16	121.6 (3)	O4—N4—C28	116.5 (5)
C16—C15—H15	119.2	O5—N4—C28	116.7 (5)
C10—C16—H16	119.7	O5—N4—O4	126.7 (6)
C15—C16—C10	120.5 (3)	O4A—N4—C28	116.5 (10)
C15—C16—H16	119.7	O4A—N4—O5A	124.0 (12)
N1—C17—C18	127.1 (2)	O5A—N4—C28	119.4 (9)
N1—C17—N2	106.0 (2)	O6—N5—C30	113.3 (5)
N2—C17—C18	126.9 (2)	O7—N5—C30	121.1 (7)
C19—C18—C17	121.3 (2)	O7—N5—O6	124.4 (8)
C19—C18—C24	118.3 (2)	O6A—N5—C30	119.8 (5)
C24—C18—C17	120.4 (2)	O6A—N5—O7A	122.4 (6)
C18—C19—H19	119.7	O7A—N5—C30	117.7 (4)
C20—C19—C18	120.6 (3)		
			
C1—C2—C3—C4	178.5 (3)	C25—C30—N5—O6	161.0 (9)
C1—C2—C8—C7	−178.5 (4)	C25—C30—N5—O7	−31.0 (17)
C1—C9—C10—C11	−28.2 (4)	C25—C30—N5—O6A	−144.8 (12)
C1—C9—C10—C16	149.3 (3)	C25—C30—N5—O7A	31.6 (7)
C1—C9—N2—C17	0.5 (3)	C26—C25—C30—C29	3.4 (4)
C2—C1—C9—C10	−5.6 (5)	C26—C25—C30—N5	−177.8 (3)
C2—C1—C9—N2	172.0 (3)	C26—C27—C28—C29	0.2 (6)
C2—C1—N1—C17	−173.2 (2)	C26—C27—C28—N4	178.1 (4)
C2—C3—C4—C5	0.1 (6)	C27—C26—N3—O2	159.3 (17)
C3—C2—C8—C7	0.4 (6)	C27—C26—N3—O3	−27.5 (11)
C3—C4—C5—C6	−179.0 (4)	C27—C26—N3—O2A	−177.8 (12)
C3—C4—C5—C7	0.1 (6)	C27—C26—N3—O3A	11 (2)
C4—C5—C7—C8	−0.2 (7)	C27—C28—C29—C30	−0.9 (6)
C5—C7—C8—C2	−0.1 (7)	C27—C28—N4—O4	13.5 (13)
C6—C5—C7—C8	179.0 (4)	C27—C28—N4—O5	−168.5 (9)
C8—C2—C3—C4	−0.4 (6)	C27—C28—N4—O4A	−32 (2)
C9—C1—C2—C3	101.6 (4)	C27—C28—N4—O5A	144.9 (19)
C9—C1—C2—C8	−79.6 (4)	C28—C29—C30—C25	−1.1 (5)
C9—C1—N1—C17	0.0 (3)	C28—C29—C30—N5	−179.9 (3)
C9—C10—C11—C12	176.3 (3)	C29—C28—N4—O4	−168.5 (11)
C9—C10—C16—C15	−176.2 (3)	C29—C28—N4—O5	9.5 (10)
C10—C9—N2—C17	178.3 (2)	C29—C28—N4—O4A	146 (2)
C10—C11—C12—C13	−0.2 (4)	C29—C28—N4—O5A	−37.1 (19)
C11—C10—C16—C15	1.4 (4)	C29—C30—N5—O6	−20.2 (10)
C11—C12—C13—C14	−177.8 (3)	C29—C30—N5—O7	147.8 (17)
C11—C12—C13—C15	1.7 (4)	C29—C30—N5—O6A	34.0 (12)
C12—C13—C15—C16	−1.7 (4)	C29—C30—N5—O7A	−149.6 (7)
C13—C15—C16—C10	0.1 (4)	C30—C25—C26—C27	−4.2 (4)
C14—C13—C15—C16	177.8 (3)	C30—C25—C26—N3	173.5 (3)
C16—C10—C11—C12	−1.3 (4)	N1—C1—C2—C3	−87.1 (4)
C17—C18—C19—C20	178.6 (3)	N1—C1—C2—C8	91.8 (4)
C17—C18—C24—C23	−177.9 (2)	N1—C1—C9—C10	−177.9 (3)
C18—C17—N1—C1	178.3 (2)	N1—C1—C9—N2	−0.3 (3)
C18—C17—N2—C9	−178.5 (2)	N1—C17—C18—C19	−176.1 (3)
C18—C19—C20—C21	−1.2 (5)	N1—C17—C18—C24	2.3 (4)
C19—C18—C24—C23	0.5 (4)	N1—C17—N2—C9	−0.4 (3)
C19—C20—C21—C22	−179.1 (3)	N2—C9—C10—C11	154.6 (3)
C19—C20—C21—C23	1.5 (5)	N2—C9—C10—C16	−27.9 (4)
C20—C21—C23—C24	−0.8 (4)	N2—C17—C18—C19	1.5 (4)
C21—C23—C24—C18	−0.2 (4)	N2—C17—C18—C24	179.9 (2)
C22—C21—C23—C24	179.8 (3)	N2—C17—N1—C1	0.2 (3)
C24—C18—C19—C20	0.2 (4)	N3—C26—C27—C28	−175.1 (3)
C25—C26—C27—C28	2.6 (5)	N4—C28—C29—C30	−178.9 (4)
C25—C26—N3—O2	−18.5 (18)	O1—C25—C26—C27	175.5 (3)
C25—C26—N3—O3	154.7 (11)	O1—C25—C26—N3	−6.9 (4)
C25—C26—N3—O2A	4.4 (13)	O1—C25—C30—C29	−176.2 (3)
C25—C26—N3—O3A	−167 (2)	O1—C25—C30—N5	2.5 (5)

### 2,4,5-Tris(4-methylphenyl)-1H-imidazol-3-ium 2,4,6-trinitrophenolate Hydrogen-bond geometry (Å, º)

**Table:**

*D*—H···*A*	*D*—H	H···*A*	*D*···*A*	*D*—H···*A*
N1—H1···O1	0.86	1.85	2.684 (3)	163
N2—H2···O2^i^	0.86	2.09	2.927 (17)	165
N2—H2···O2*A*^i^	0.86	2.16	2.974 (11)	158

Symmetry code: (i) *x*, −*y*+1/2, *z*+1/2.
